# Deficiency of Interleukin-15 Enhances Susceptibility to Acetaminophen-Induced Liver Injury in Mice

**DOI:** 10.1371/journal.pone.0044880

**Published:** 2012-09-18

**Authors:** Hsein-San Hou, Ching-Len Liao, Huey-Kang Sytwu, Nan-Shih Liao, Tien-Yu Huang, Tsai-Yuan Hsieh, Heng-Cheng Chu

**Affiliations:** 1 Graduate Institute of Life Sciences, National Defense Medical Center, Taipei, Taiwan; 2 Department of Microbiology and Immunology, National Defense Medical Center, Taipei, Taiwan; 3 Institute of Molecular Biology, Academia Sinica, Taipei, Taiwan; 4 Division of Gastroenterology and Hepatology, Department of Internal Medicine, Tri-Service General Hospital, National Defense Medical Center, Taipei, Taiwan; National Institutes of Health, United States of America

## Abstract

Hepatocytes have a direct necrotic role in acetaminophen (APAP)-induced liver injury (AILI), prolonged secondary inflammatory response through innate immune cells and cytokines also significantly contributes to APAP hepatotoxicity. Interleukin 15 (IL-15), a multifunction cytokine, regulates the adaptive immune system and influences development and function of innate immune cells. To better understand the role of IL-15 in liver injury, we treated wild-type (WT) and IL-15-knockout (*Il15^−/−^*) mice with a hepatotoxic dose of APAP to induce AILI and evaluated animal survival, liver damage, APAP metabolism in livers and the inflammatory response. Production of pro-inflammatory cytokines/chemokines was greater in *Il15^−/−^* than WT mice. Subanalysis of hepatic infiltrated monocytes revealed greater neutrophil influx, along with greater hepatic induction of inducible nitric oxide synthase (iNOS), in *Il15^−/−^* than WT mice. In addition, the level of hepatic hemeoxygenase 1 (HO-1) was partially suppressed in *Il15^−/−^* mice, but not in WT mice. Interestingly, elimination of Kupffer cells and neutrophils did not alter the vulnerability to excess APAP in *Il15^−/−^* mice. However, injection of galactosamine, a hepatic transcription inhibitor, significantly reduced the increased APAP sensitivity in *Il15^−/−^* mice but had minor effect on WT mice. We demonstrated that deficiency of IL-15 increased mouse susceptibility to AILI. Moreover, Kupffer cell might affect APAP hepatotoxicity through IL-15.

## Introduction

Acetaminophen (APAP) is an over-the-counter analgesic widely used worldwide. However, APAP-induced liver injury (AILI) represents the most common hepatogenous poisoning secondary to drug overdose. Excess APAP saturates the sulfation and glucuronidation of the metabolic pathway and results in generation of toxic N-acetyl-p-benzoquinone imine (NAPQI) by cytochrome P450 (CYP) [1], thereby depleting hepatic glutathione (GSH) [2]. Residual unconjugated NAPQI induces covalent binding of intracellular proteins and causes further formation of reactive oxygen species (ROS) [3], thus resulting in apoptosis and necrosis of hepatocytes [4]. Induction of intracellular inflammation regulatory proteins such as hemeoxygenase 1 (HO-1) attenuates APAP toxicity [5]. In addition, the downstream innate immune response, by immune cells and associated cytokines, modulates the progression of liver injury [6].

Innate immune cells such as natural killer (NK) cells, natural killer T (NKT) cells [7], neutrophils [8], dendritic cells (DCs) [9], and Kupffer cells (KCs) [10], [11] play important roles in AILI. Depletion of NK and NKT cells by an antibody retarded APAP toxicity in mouse liver [7]. However, Masson *et al.* showed an indefinite role of NK and NKT cells in AILI [12]. The uncertain role of neutrophils in AILI was shown in different studies [8], [13]. Recently, increased APAP sensitivity was attributed to enhanced inflammation in mice lacking DCs, but the detailed mechanism remained speculative [9]. Depletion or inactivation of KCs by chemicals in an AILI model had controversial results, with a protective effect in one study [11] but a negative result in another [10]. Furthermore, mice lacking of cytokines such as interleukin 10 (IL-10) [14], IL-6 [15] or IL-13 [16] were found susceptible to APAP hepatotoxicity, whereas induction of pro-inflammatory mediators such as tumor necrosis factor alpha (TNFα) [17], interferon gamma (IFNγ) [18], IL-18 or IL-1β [19] and nitric oxide (NO) enhanced AILI in mice. Collectively, the roles of innate immune cells, especially antigen-presenting cells, and cytokines in AILI are complicated and still unclear.

IL-15, a multifunction cytokine mainly produced by antigen-presenting cells such as macrophages, DCs, B cells or endothelial cells, regulates the adaptive immune system and plays an important role in innate immunity [20], [21]. IL-15 can direct the development of CD8+ memory T cells, NK and NKT cells [20] and modulate the function of macrophages and DCs [22]. In addition, IL-15 can inhibit apoptosis of neutrophils [23] and regulate the production of inflammatory cytokines such as TNFα, IL-6, IL-1β and IL-10 in macrophages in response to lipopolysaccharide stimulation [24]. Synthetic IL-15 could moderately diminish liver injury in concanavalin A or Fas ligand-induced hepatitis [25], [26], whereas DC-derived IL-15 enhanced endotoxin shock injury through the liver [27]. Moreover, IL-15 mediates the crosstalk between conventional and plasmacytoid DCs for immune activation [28]. Interestingly, IL-15 promoted hepatocyte mitosis and liver proliferation in healthy mice and those with hepatectomy, respectively [29].

In this study, we aimed to study the role of IL-15 in a sterile APAP-induced fulminant hepatitis model in IL-15-knockout (*Il15^−/−^*) and wild-type (WT) mice. By treatment of mice with a hepatotoxic dose of APAP and evaluation of animal survival, liver damage, APAP metabolism in livers and the inflammatory response, we were able to elucidate whether IL-15 was involved in APAP hepatitis.

## Materials and Methods

### Animals and materials

Male C57BL/6J mice were purchased from National Laboratory Animal Center, Taipei, Taiwan. C57BL/6NTac/*Il15^−/−^* mice, obtained from Taconic Farms (Terrytown, NY), were backcrossed to C57BL/6J background for 4 generations. This substrain, as C57BL/6J/*Il15^−/−^* (presented as *Il15^−/−^* in our study) mouse, was used in our later study. All mice (9–12 weeks old) were kept in a pathogen-free condition in compliance with institutional animal care and use committee guidelines (project I.D., IACUC NO. 10-043). All chemicals were purchased from Sigma Chemical Co. (St. Louis, MO, USA) unless otherwise stated. Recombinant murine IL-15, with specific activity of ≥2×10^5^ units/mg, was purchased from Perprotech (Rocky Hill, NJ, USA). Rat anti-IL-15 neutralizing antibody was obtained from eBiscience (San Diego, CA, USA). Recombinant IL-15 was intraperitoneally or subcutaneously injected 20 minutes before APAP challenge. IL-15 neutralizing antibody was intraperitoneally injected 30 minutes prior to APAP administration. APAP was dissolved in warm saline and intraperitoneally injected into overnight-fasted mice to induce hepatitis, and 350 mg/kg of APAP (sub-lethal dose) were generally used in this study unless otherwise mentioned. Animal survival was monitored for 5 days. 1.5 mg/kg of vinblastine (vin) or 30 mg/kg of gadolinium chloride (GdCl_3_) was intravenously given to animals for neutrophil or KC elimination, respectively.

### Evaluation of liver injury

Serum alanine and aspartate aminotransferase (ALT/AST) levels were determined by the test kit DRY-CHEM SLIDE ALT/AST (Fujifilm, Tokyo, Japan). The liver was sectioned and fixed overnight in 10% formalin solution, dehydrated, paraffin-embedded, cut at 4-mm thickness and stained with haematoxylin and eosin (H&E) for histological examination. The liver necrotic area was quantified by 2 individual pathologists with Image-Pro Plus (Media Cybernetics, Bethesda, MD) and the necrotic area (%) was expressed as necrosis area/total area of liver section.

### Hepatocyte isolation and culture, and KC-enriched fractionation

Hepatocytes were isolated from mice by a 2-step collagenase perfusion protocol. The hepatic portal vein was ligated and perfused with Ca^2+^/Mg^2+^-free Hank's balanced salts solution (HBSS; Gibco, Invitrogen, Carlsbad, CA) with 5 mM EGTA, than 0.025% type IV collagenase containing HBSS. After perfusion, liver was excised and hepatocytes were suspended in serum free L-15 medium and pass through 100 mm strainer. The filtrate was centrifuged at 45 g for 2 min at 4°C. The supernatant as the non-parenchymal cell (NPC) fraction was centrifuged at 350 g for 5 min at 4°C and, used to analyze mononuclear cell population or KC enrichment.

After perfusion, hepatocyte viability (more than 85%) was determined by trypan blue exclusion. Hepatocytes were suspended in 10% fetal bovine serum containing William's Essential Medium (WEM) and cultured in 0.02% collagen-coated plate; after 4 hrs, the indicated concentration of APAP was added to analyze cytotoxicity to hepatocytes. The APAP toxicity was quantified by use of the Cell Counting Kit-8 (CCK-8).

NPCs underwent a 25%/50% two-step Percoll gradient to isolated KC-enriched cells.

### Hepatic glutathione (GSH) measurement

Hepatic total GSH was measured by 5,5′-dithiobis(2-nitrobenzoic acid) (DTNB) assay. Frozen liver samples were homogenized with 4 volumes (v/w) of 5% trichloroacetic acid, and supernatant was incubated with DTNB for 15 min. Hepatic GSH concentration was measured by colorimetry at optical density 412 nm.

### Determination of cytokines by ELISA

Murine IL-15, IL-1β, IFNγ and TNFα levels in serums or livers were measured by ELISA kits (R&D research). Serum samples were collected by heart puncture and liver proteins were collected from frozen liver samples homogenized in protein lysis buffer.

### Immunohistochemical staining of liver samples

The levels of hepatic inducible NO synthase (iNOS) and nitrotyrosine were evaluated in deparaffinized and antigen-retrieved liver sections by LSAB2 system-HRP kits (DakoCytomation, Produktionsvej, Denmark). Liver sections were incubated with primary antibodies against iNOS or nitrotyrosine (Upstate Biotechnology, Lake Placid, NY) as the manufacture's instructions. Immunohistochemical staining was analyzed by use of Image-Pro Plus (Media Cybernetics, Bethesda, MD), with positive area (%) = positive signal area/total area of liver section.

### Western blot analysis

The snap-frozen liver samples were thawed on ice, then homogenization with 9 volumes (v/w) of protein lysis buffer [25 mM HEPES (pH 7.3) containing 5 mM EDTA, 5 mM DTT, 0.1% CHAPS and protease inhibitor cocktail (Roche, Penzberg, Germany)]. Protein underwent SDS-PAGE, then were transferred to PVDF membranes (Millipore, Billerica, MA), which were incubated with antibodies against HO-1 (Epitomics, Inc., Burlingame, CA), CYP2E1 (Abcam, Cambridge, UK), CYP1A2, glutamate-cysteine ligase, catalytic subunit (GCLC), and heat shock protein 70 (Hsp70/HSC70; Santa Cruz Biotechnology, Santa Cruz, CA). Target signals were detected by use of enhanced chemiluminescence (Millipore, Billerica, MA) and LAS-3000 (Fujifilm, Tokyo, Japan). The relative protein expression was quantified by densitometry with Image J (NIH).

### Real-time PCR

Hepatic RNA was isolated by Trizol reagent (Gibco, Invitrogen, Carlsbad, CA) from ∼50 mg of liver sample. cDNA was reversely transcribed by the H minus reverse transcription kit (Fermentas, Thermo Fisher Scientific, Waltham, MA). Total 0.05 µg RNA-transcripted cDNA was processed by the SYBR green method with ABI-7500 (Applied Biosystems, Inc., Foster city, CA). The primer sequences for target genes are listed in Table S1. The expression of target genes was normalized to that of glyceraldehyde 3-phosphate dehydrogenase (GAPDH) and calculated relative to the control.

### Statistical analysis

Data are presented as mean ± SEM. Statistical significance was determined by the Student's t test and the log-rank test by use of GraphPad Prism 5 (GraphPad Software, La Jolla, CA). *P*<0.05 was considered statistically significant. (See Additional Supporting Methods S1.)

## Results

### Deficiency of IL-15 enhanced susceptibility to APAP-induced fulminant hepatitis in mice

**Figure 1:**
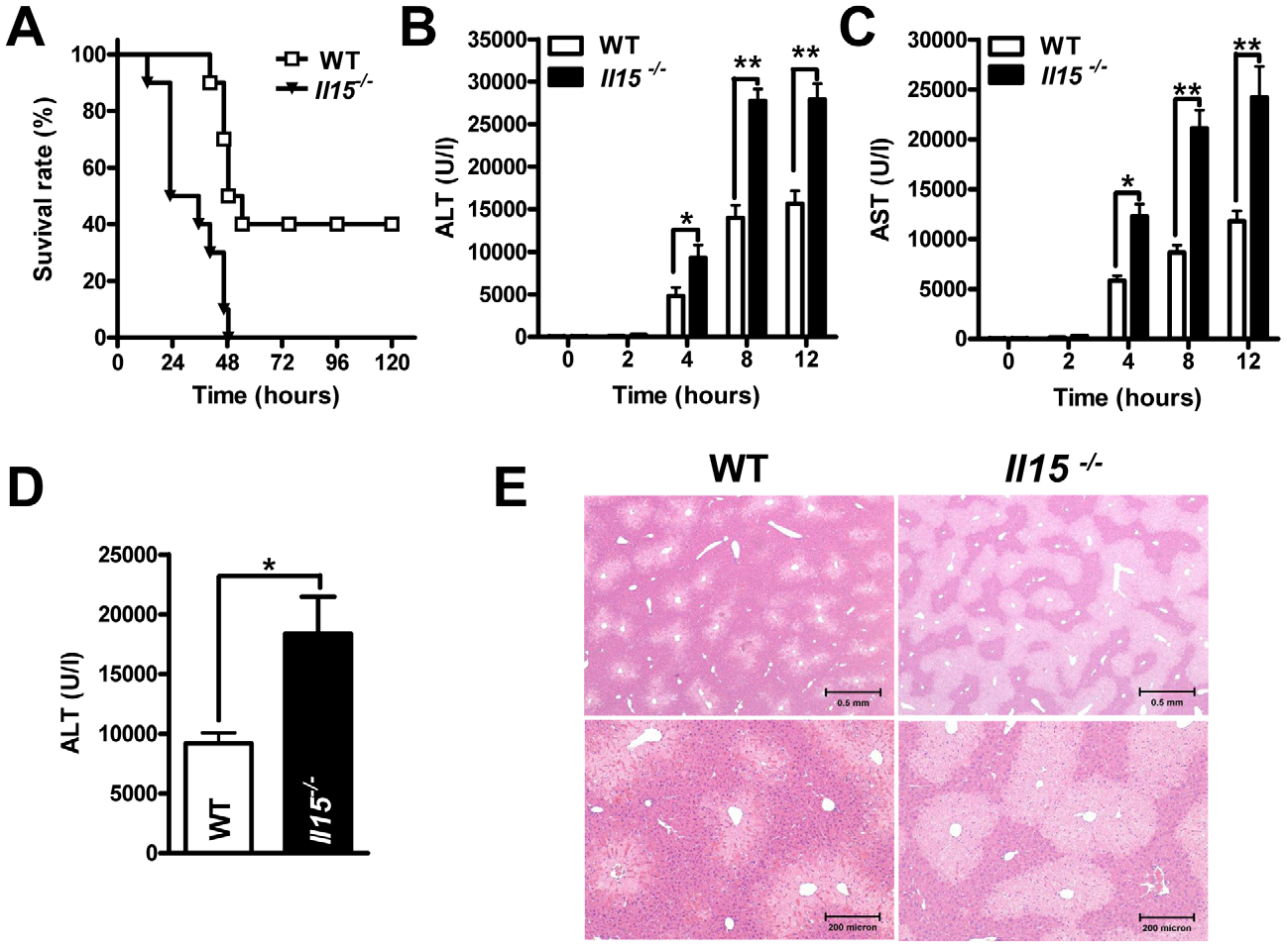
Deficiency of IL-15 exacerbates APAP-induced fulminant hepatitis in mice. (A) Survival of *Il15^−/−^* (n = 8) and WT (n = 10) mice for 5 days after APAP injection. *P*<0.01 for *Il15^−/−^* mice compared to WT controls. Serum levels of (B) ALT and (C) AST at indicated times after treatment with APAP. (D) Serum ALT levels and (E) liver histopathological changes at 8 hr after treatment with 200 mg/kg of APAP (H&E staining; magnification, upper panels, ×4; lower panel, ×10). *P<0.05; **P<0.001. Data are mean ± SEM from 5∼8 mice per group.

Although IL-15 was previously shown to attenuate Jo-2 and concanavalin A-induced liver injuries [25], [26], its role in drug-induced liver injury remained elusive. In our model of hepatitis induced by 400 mg/kg (lethal dose) of APAP in mice, the 5-day mortality was higher for *Il15^−/−^* than WT mice [100% (8/8) vs. 60% (6/10)] (Figure 1A). To confirm this result, we examined the serum activities of the liver enzymes ALT and AST in *Il15^−/−^* and WT mice. Four hours after APAP injection, the enzyme levels significantly increased and peaked between 8 and 12 hours post-APAP injection in *Il15^−/−^* mice, as compared to WT counterparts (Figure 1B,C). Another non-lethal dose (200 mg/kg) of APAP was further given to demonstrate this increased APAP sensitivity in *Il15^−/−^* mice. Compared with WT controls, *Il15^−/−^* mice showed higher serum ALT levels at 8 hr post-APAP injection (Figure 1D), which were further confirmed by histopathological examination of damaged livers, with more extensive centrilobular necrosis in *Il15^−/−^* mice (Figure 1E). Thus, deficiency of IL-15 increased the susceptibility to AILI in mice.

### IL-15 was induced in the KC-enriched fraction in APAP-induced hepatic injury

**Figure 2:**
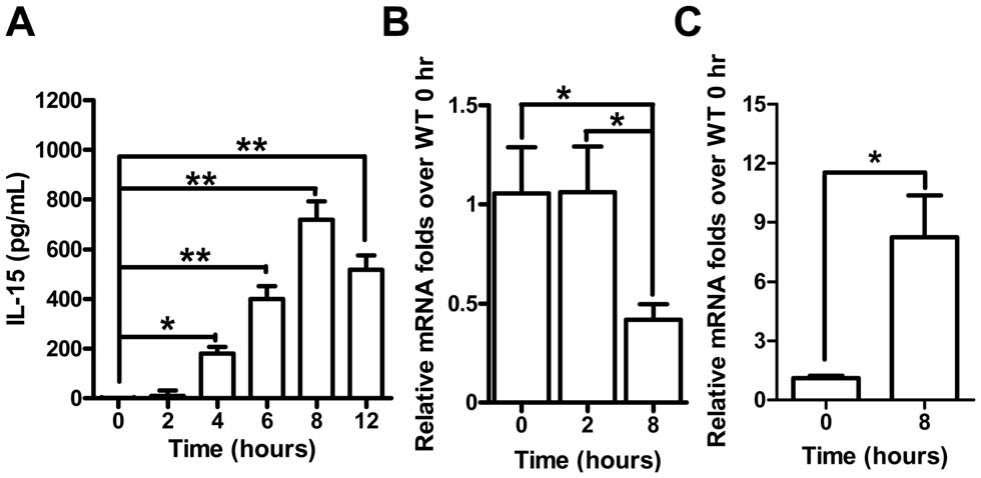
IL-15 was elevated in serum and up-regulated in KC-enriched fraction but not in total liver during AILI in WT mice. (A) IL-15 levels in mouse serum at indicated time points after APAP injection were evaluated by ELISA. Relative mRNA levels of IL-15 in (B) total liver at 0, 2 and 8 hr, and (C) KC-enriched fraction at 0 and 8 hr after APAP injection were analyzed by quantitative PCR. *P<0.05; **P<0.01. Data are mean ± SEM from 6∼8 mice per group.

Because of enhanced APAP hepatotoxicity in *Il15^−/−^* mice, we further examined IL-15 serum levels and hepatic expression in mice. The serum IL-15 levels significantly increased at 4 hr and reached to peak at 8 hr post-APAP in WT mice (Figure 2A), which were in parallel to liver damages by ALT levels. In contrast, expression of hepatic IL-15 was reduced at 8 but not 2 hr post-APAP in WT mice (Figure 2B), indicating IL-15 was not up-regulated in hepatic parenchymal cells during AILI. Monocytes (e.g., KCs) were previously reported to be the dominant IL-15-producing cells [30]. Hence, we examined the expression of IL-15 in the KC-enriched cell fraction. WT mice showed 7- to 8-fold increased mRNA expressions of IL-15 at 8 hr after APAP injection (Figure 2C). Thus, IL-15 was predominantly up-regulated in KCs but not in hepatocytes during AILI.

### Enhanced susceptibility to AILI in *Il15^−/−^* mice is not mediated by altered metabolism and ROS detoxification of APAP in hepatocytes

**Figure 3:**
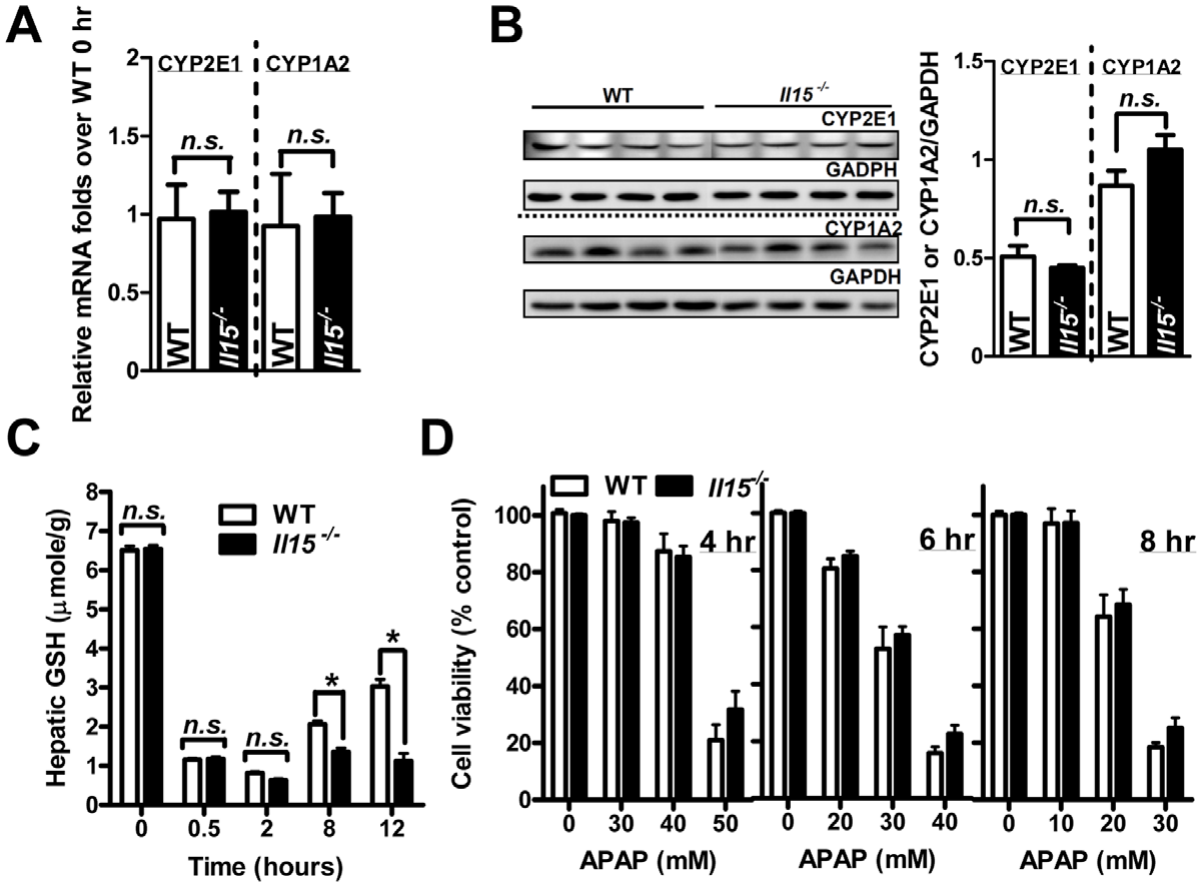
Hepatic metabolism of APAP does not contribute to the enhanced susceptibility to AILI in *Il15^−/−^* mice. The changes in APAP metabolic enzyme levels by analysis of hepatic mRNA and protein levels of CYP2E1 and CYP1A2 by (A) quantitative PCR and (B) western blot analysis and quantification. GAPDH served as the loading control. (C) Hepatic GSH levels at 0, 0.5, 2, 8 and 12 hr after APAP treatment. (D) Viability of isolated primary hepatocytes after treatment with 0 to 50 mM of APAP for 4 to 8 hr. *P<0.05; *n.s.*, not significant. Data are mean ± SEM from 7∼8 mice per group.

To exclude that APAP metabolites participate in the heightened APAP hepatotoxicity in *Il15^−/−^* mice, we examined the expressions of CYP2E1 and CYP1A2, the main enzymes to oxidize excess APAP to toxic NAPQI [1] in liver. Hepatic CYP2E1 and CYP1A2 expressions were similar in WT and *Il15^−/−^* mice at 0 hr (Figure 3A,B). Additionally, GSH depletion in liver indirectly reflects the elimination of APAP metabolites. Both groups of mice showed similar GSH levels at 0.5 and 2 hr, with the lowest GSH level at 2 hr post-APAP (Figure 3C). Interestingly, delayed GSH recovery was observed at 8 and 12 hr post-APAP in *Il15^−/−^* mice but not in WT mice. We further analyzed *in vitro* APAP sensitivity of isolated hepatocytes from individual mice. The hepatocyte viability after 4, 6 and 8 hrs of APAP treatment did not differ between WT and *Il15^−/−^* mice (Figure 3D).

**Figure 4:**
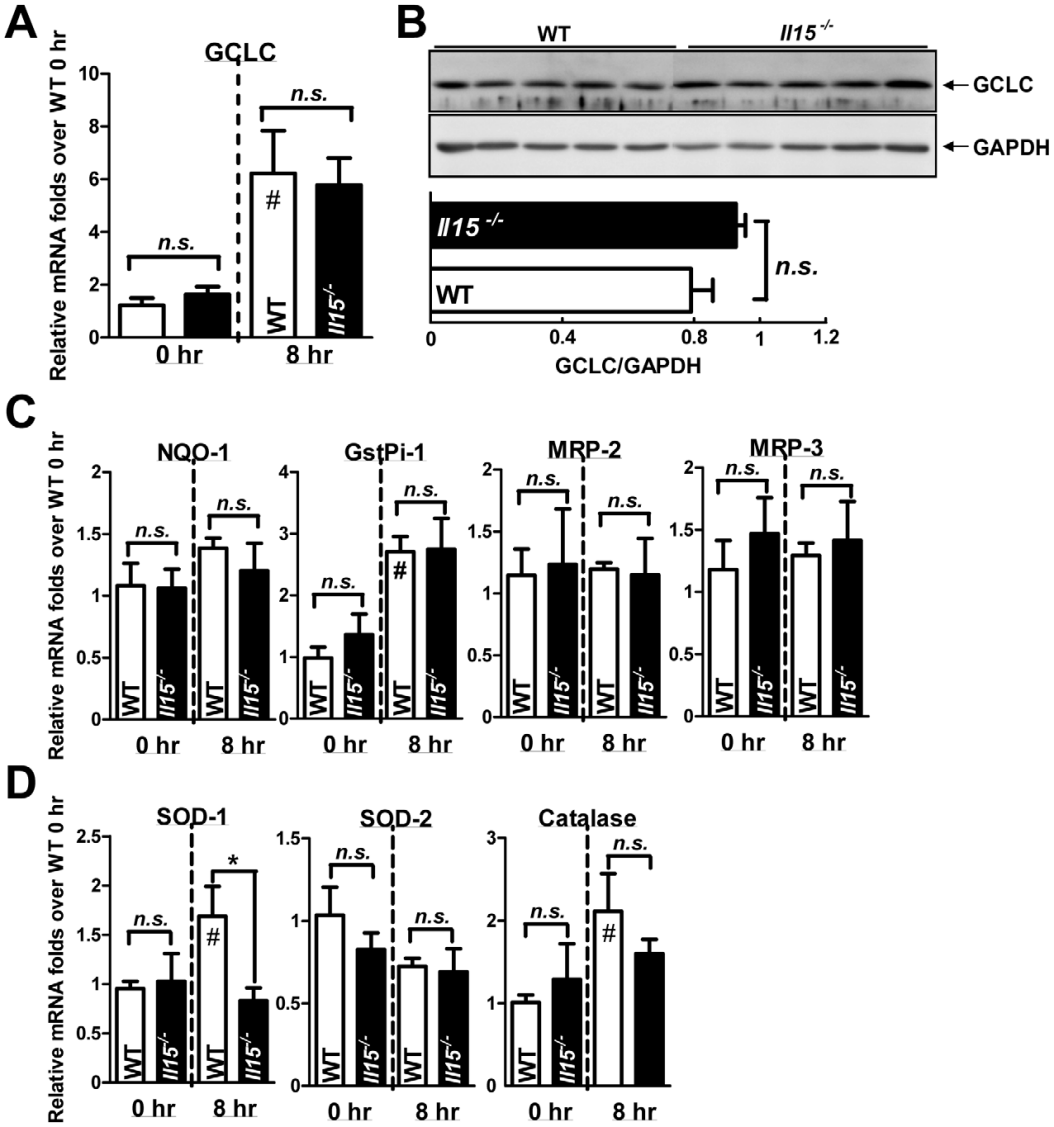
The similar activation of hepatic Nrf2-related and ROS detoxification genes in APAP-injected mice. (A) The hepatic mRNA and (B) protein levels of GCLC at 0 and 8 hr after APAP challenge; representative and quantitative data are in upper and lower panels, respectively. Hepatic mRNA levels of Nrf2-related genes (C) NQO-1, GstPi-1, MRP-2, and MRP-3, and ROS detoxification genes (D) SOD-1, SOD-2 and catalase at 0 and 8 hr after APAP challenge. *P<0.05; #*P*<0.05 compared with WT at 0 hr; *n.s.*, not significant. Data are mean ± SEM from 7∼8 mice per group in (A), and 4∼5 mice per group in (B).

To explore the causes of diminished GSH recovery in *Il15^−/−^* mice, we determined synthetic efficiency of GSH by analyzing GCLC expression [31]. However, GCLC levels did not differ in both groups of mice at 0 and 8 hr post-APAP (Figure 4A,B). This finding indicated the delayed GSH recovery in *Il15^−/−^* mice was probably attributed to the less residual functional hepatocytes after APAP injection. Nuclear factor erythroid 2-related factor 2 (Nrf2)-related and ROS detoxification genes also mediate the sensitivity of APAP hepatotoxicity [32], [33]. Therefore, we further examined the activation of Nrf2-related genes, such as NAD(P)H:quinone oxidoreductase 1 (NQO-1), glutathione S-transferase pi 1 (GstPi-1), multidrug resistance protein 2 (MRP-2), MRP-3, etc. and ROS detoxification genes, such as superoxide dismutase-1 (SOD-1), superoxide dismutase-2 (SOD-2), catalase, etc. Except for the minimal suppression of SOD-1 in *Il15^−/−^* mice, both groups did not differ in mRNA expression levels of these genes (Figure 4C,D). Thus, metabolism of APAP and induction of ROS detoxification genes in hepatocytes do not contribute to the heightened APAP sensitivity in *Il15^−/−^* mice.

### APAP treatment induced a stronger inflammatory response in *Il15^−/−^* mice than WT mice

**Figure 5:**
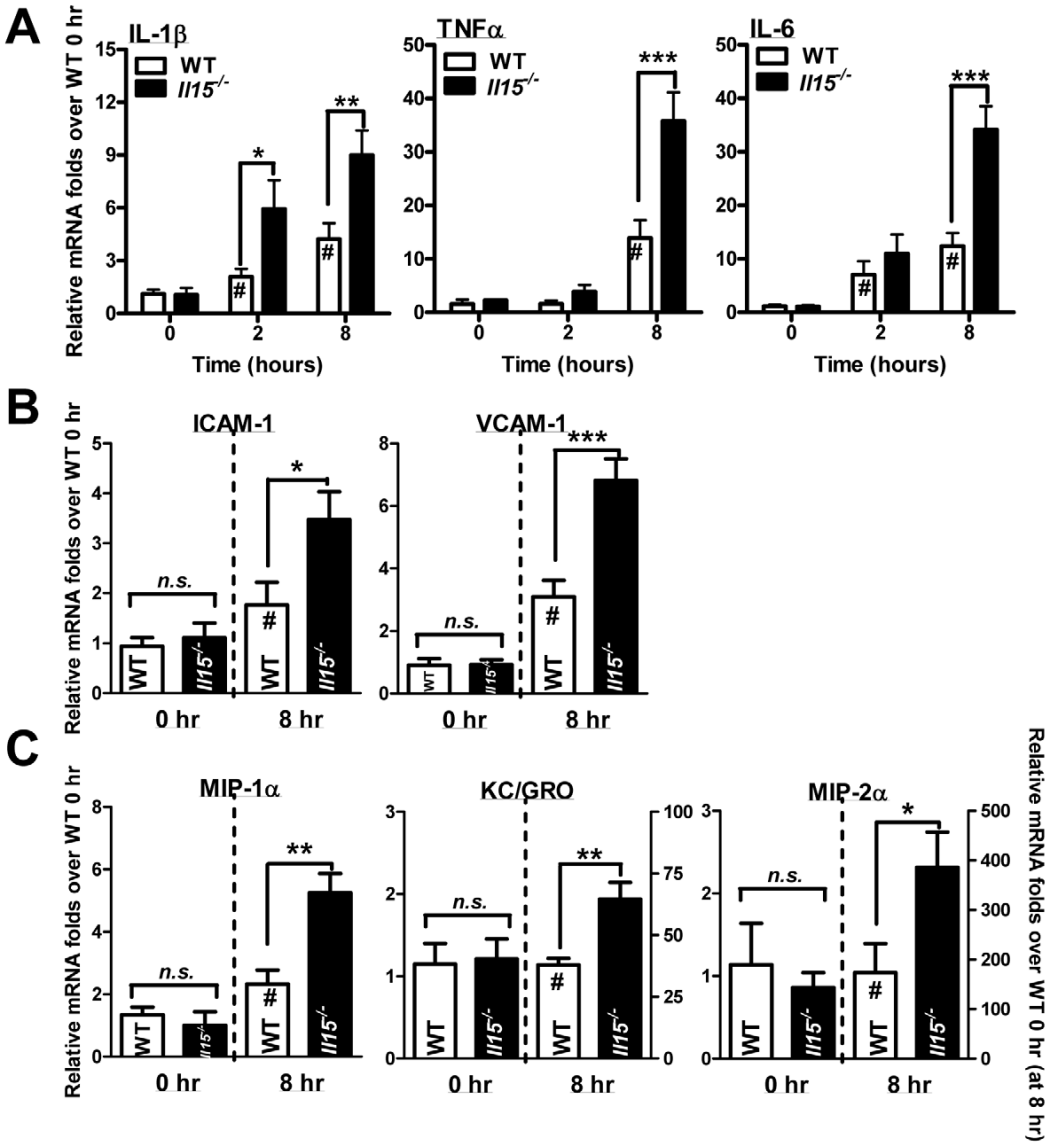
Inductions of hepatic inflammatory genes are greater in *Il15^−/−^* than WT mice with APAP challenge. Relative mRNA levels of (A) pro-inflammatory cytokines IL-1β, TNFα and IL-6; (B) vascular adhesion molecules ICAM-1 and VCAM-1; and (C) chemokines MIP-1α, KC/GRO and MIP-2α at indicated times after injection with APAP. *P<0.05; **P<0.01; ***P<0.001; #*P*<0.05 compared with WT at 0 hr. Data are mean ± SEM from 6∼8 mice per group.

Because inflammation caused by necrotic hepatocytes participated in AILI [6], we further examined the APAP-induced inflammatory response in mice. The mRNA levels of hepatic pro-inflammatory cytokines IL-1β, TNFα and IL-6 were higher at 8 hr post-APAP in *Il15^−/−^* mice than WT counterparts, with a higher IL-1β level at 2 hr (Figure 5A). Moreover, the serum levels of IL-1β, TNFα and IFNγ and liver levels of IL-1β and IFNγ were also higher in *Il15^−/−^* mice than WT controls (Figure S1). *Il15^−/−^* mice showed stronger induction of the adhesion molecules, intercellular adhesion molecule-1 (ICAM-1) and vascular cell adhesion protein-1 (VCAM-1) as well as neutrophilic chemokines, such as macrophage inflammatory protein-1 alpha (MIP-1α), KC/GRO and MIP-2α at 8 hr post-APAP (Figure 5B,C).

Because of the enhanced production of hepatic neutrophilic chemokines in *Il15^−/−^* mice, we investigated the profile of infiltrated monocytes in mouse livers. NPCs with CD11b^high^F4/80^high^ expression were referred to KCs [34], and the population of CD11b^high^F4/80^low^ cells were confirmed to be neutrophils by anti-Gr-1 antibody (data not shown) [34]. In the resting state, >90% and <4% of hepatic monocytes were KCs and neutrophils, respectively. APAP-induced neutrophil infiltration of liver was greater in *Il15^−/−^* than WT mice at 8 hr after challenge, while the infiltration of KCs was relatively lesser (Figure S2). Therefore, APAP treatment increased inflammatory cytokine production and the extent of inflammatory cell infiltration in livers of *Il15^−/−^* mice.

### APAP-injected *Il15^−/−^* mice showed discordant induction of hepatic iNOS and HO-1

**Figure 6:**
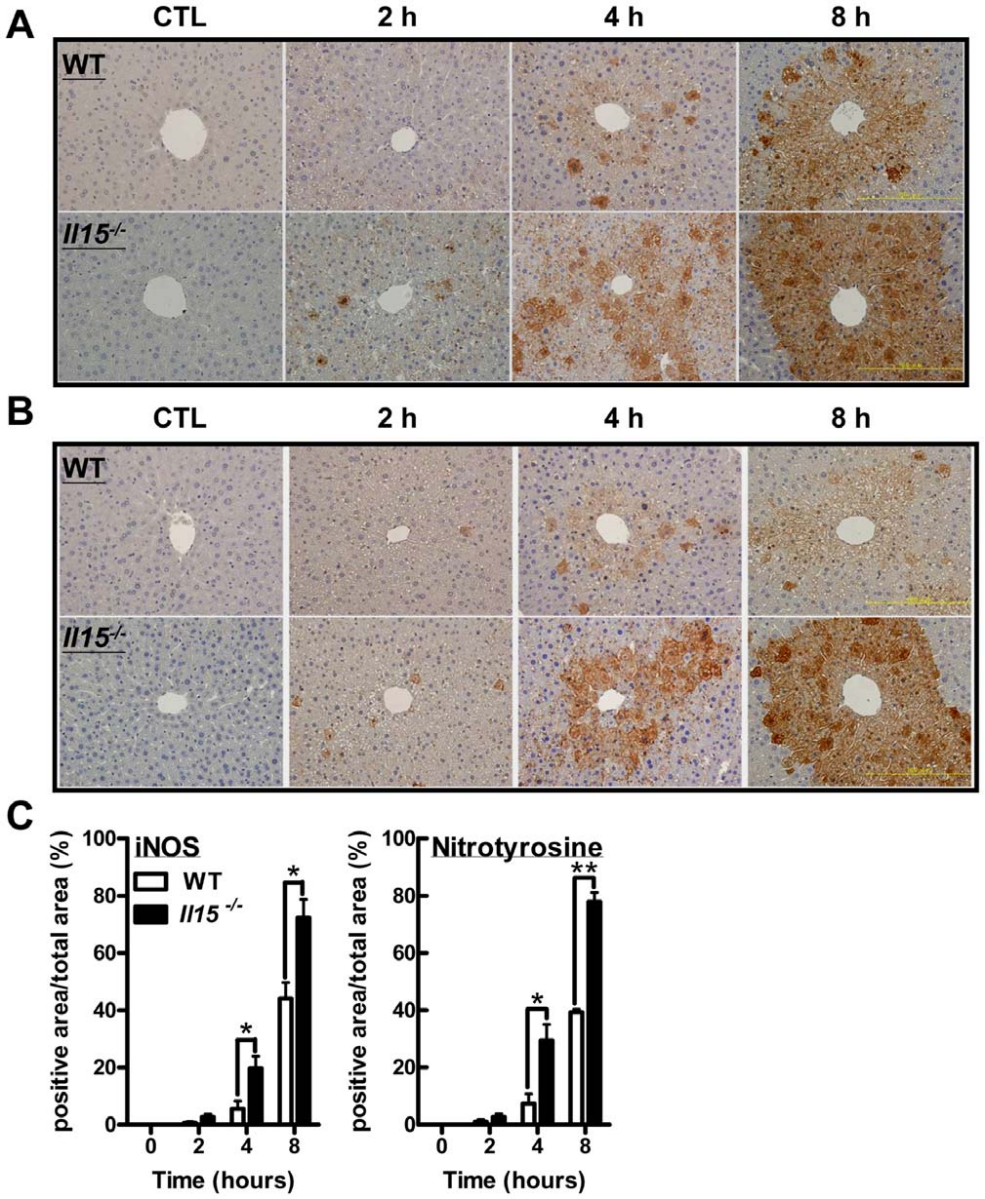
The induction of hepatic reactive nitrogen species (RNS) is greater in *Il15^−/−^* than WT mice with APAP challenge. (A) Immunohistochemistry of iNOS expression, (B) nitrotyrosine formation and (C) percentage of iNOS or nitrotyrosine-positive area at 0, 2, 4 and 8 hr after APAP challenge. *P<0.05; **P<0.01. Data are mean ± SEM from 3 mice per group.

It has been reported that, NO, a product of iNOS, contributes to the APAP-induced hepatotoxicity [35]. We therefore tested hepatic iNOS induction by immunostaining in APAP-treated mice. The *Il15^−/−^* mouse livers showed stronger iNOS inductions than those of WT livers at 4 and 8 hr post-APAP injection (Figure 6A,C), with a similar trend of nitrotyrosine accumulation in both groups of mice (Figure 6B,C). Collectively, APAP-induced reactive nitrogen species (RNS) formation was more pronounced in *Il15^−/−^* than WT mice.

**Figure 7:**
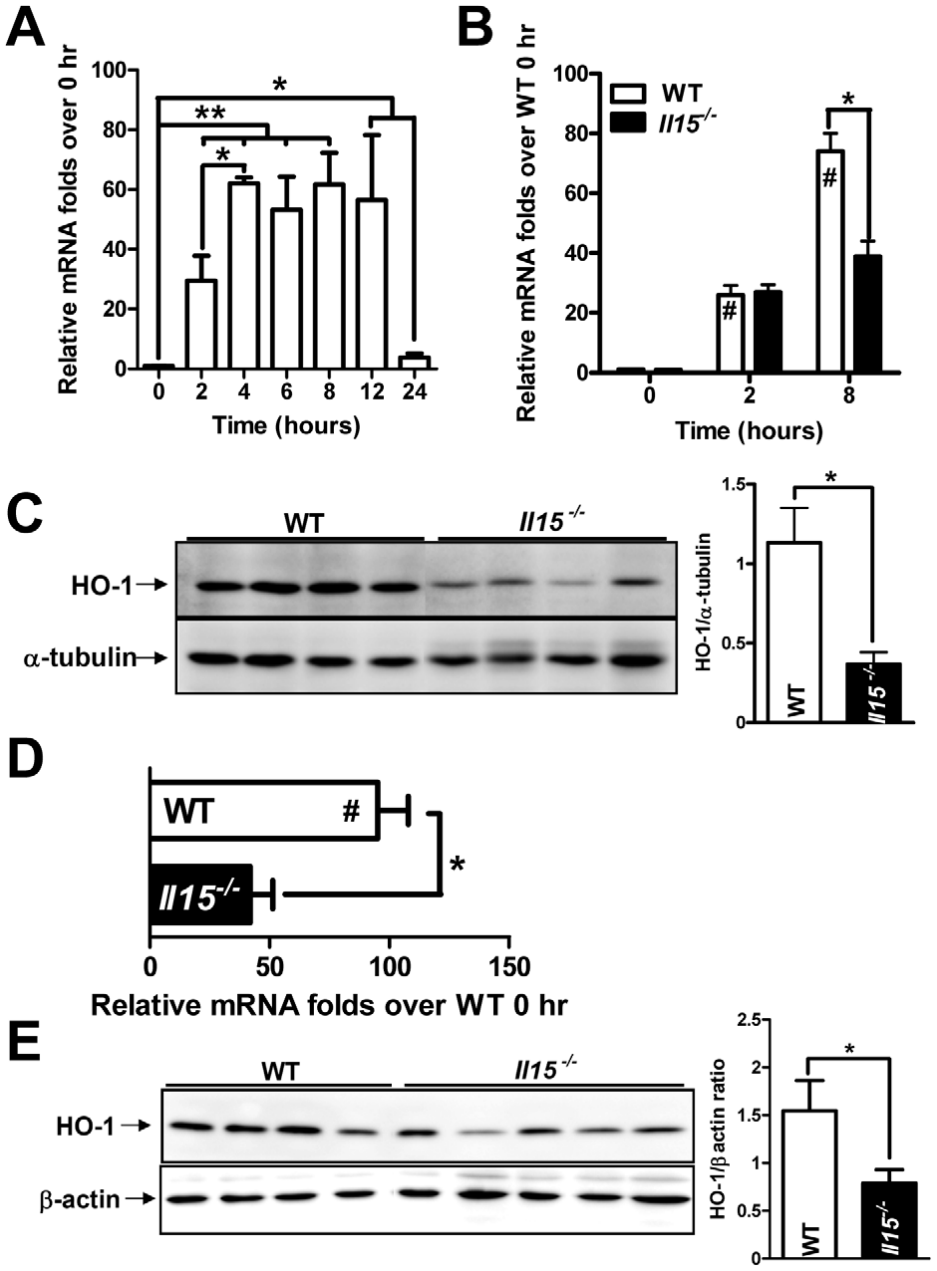
The hepatic HO-1 induction was suppressed in *Il15^−/−^* than WT mice with APAP challenge. (A) Time course of hepatic HO-1 induction after APAP injection in WT mice. HO-1 (B) mRNAs at 0, 2 and 8 hr and (C) proteins-representative and quantitative data at 8 hr post-APAP treatment. HO-1 (D) mRNA levels and (E) protein-representative and quantitative data of hepatocytes at 8 hr with APAP challenge. #*P*<0.05, compared with WT 0 hr; *P<0.05; **P<0.01; ***P<0.001. Data are mean ± SEM from 3∼6 mice per group in (A) and 6∼8 mice per group in (B∼E).

HO-1, also known as Hsp32, had been reported to protect against AILI [5]. After APAP treatment, hepatic HO-1 mRNA was induced within 2 hr and peaked at 4 hr, then returned to normal after 24 hr post-APAP in WT mice (Figure 7A). Despite similar hepatic HO-1 mRNA levels at 2 hr post-APAP in WT and *Il15^−/−^* mice, hepatic HO-1 levels were suppressed at 8 hr post-APAP in *Il15^−/−^* mice (Figure 7B). This finding was further confirmed by decreased HO-1 protein expression in livers of *Il15^−/−^* mice at the same time (Figure 7C). Previously, HO-1 was predominantly induced in parenchymal cells after APAP injection in rat [5]. Compared with WT counterparts, *Il15^−/−^* mice showed suppressed induction of HO-1 in hepatocytes (Figure 7D,E). Likewise, Hsp70 induction protected against APAP hepatitis in mice [36]. In our study, both mRNA and protein levels of hepatic Hsp70 were lesser in *Il15^−/−^* than WT mice after APAP injection (Figure S3).

### Increased susceptibility to AILI is abrogated by liver-specific transcriptional inhibitor in mice

**Figure 8:**
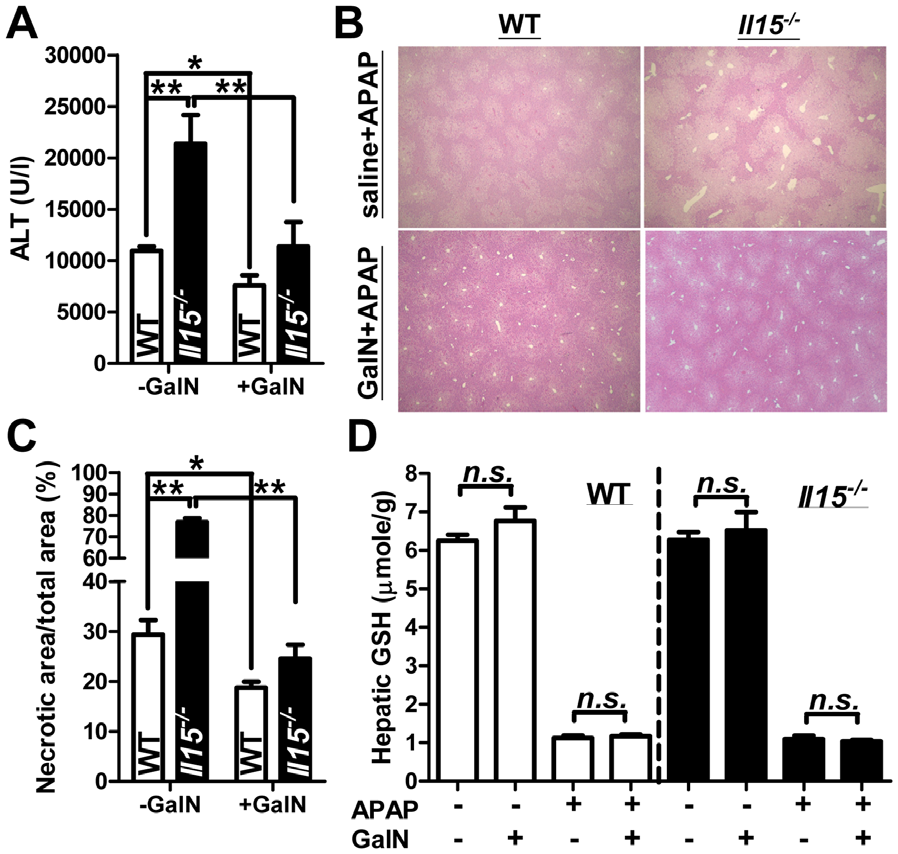
Injection of GalN reduces the enhanced susceptibility to AILI in *Il15^−/−^* and WT mice. Mice were pre-treated with or without 700 mg/kg of GalN 20 minutes before APAP challenge. (A) ALT levels in serum and (B) liver histopathological changes at 6 hr after APAP (H&E staining; magnification ×4). (C) The quantification results of hepatic necrotic area by software Image-Pro Plus analysis. (D) Hepatic GSH levels at 0.5 hr with or without APAP injection. *P<0.05; **P<0.01; *n.s.*, not significant. Data are mean ± SEM from 4∼6 mice per group.

Pro-inflammatory cytokines such as IL1-β, TNFα and IFNγ induce iNOS expressions in hepatocytes [37]. To further clarify the effecter cells in heightened APAP hepatitis of *Il15^−/−^* mice, galactosamine (GalN) was given to mice for specific inhibition of hepatocyte transcription [38]. Unexpectedly, pre-treatment with GalN abrogated the increased hepatotoxicity in *Il15^−/−^* mice in terms of serum ALT level at 6 hr post-APAP, with a minor effect on WT counterparts (Figure 8A, B & C). The reduced APAP hepatitis in *Il15^−/−^* mice, by GalN pre-treatment, was further confirmed by the serum ALT levels at 4 hr (APAP+GalN: 4250±580 U/l, APAP+saline 11250±2038 U/l, *P*<0.05) and 8 hr (APAP+GalN: 17010±1493 U/l, APAP+saline: 29860±4273 U/l, *P*<0.05), respectively. However, the GalN reduction effect on APAP hepatitis in WT mice was not as much as that in *Il15^−/−^* counterparts. With the histopathological examination and liver necrotic quantization, GalN pre-treatment reduced the APAP-related extensive cell-content release of the injured hepatocytes in *Il15^−/−^* mice (Figure 8B,C). Similar GSH depletions at 0.5 hr post-APAP were observed in WT and *Il15^−/−^* mice, with or without GalN pre-treatment (Figure 8D), which further indicated APAP metabolism, was not affected by GalN pre-treatment. Moreover, without APAP challenge, GalN pre-treatment did not affect hepatic GSH levels in WT and *Il15^−/−^* mice. Thus, heightened susceptibility to AILI in *Il15^−/−^* mice can be ameliorated by GalN pre-treatment and this effect seems to be not through alteration of hepatic GSH contents at early stage of APAP hepatitis.

### Injection of recombinant IL-15 into *Il15^−/−^* mice or IL-15 neutralizing antibody into WT counterparts did not alter the susceptibility of animals to APAP

**Figure 9:**
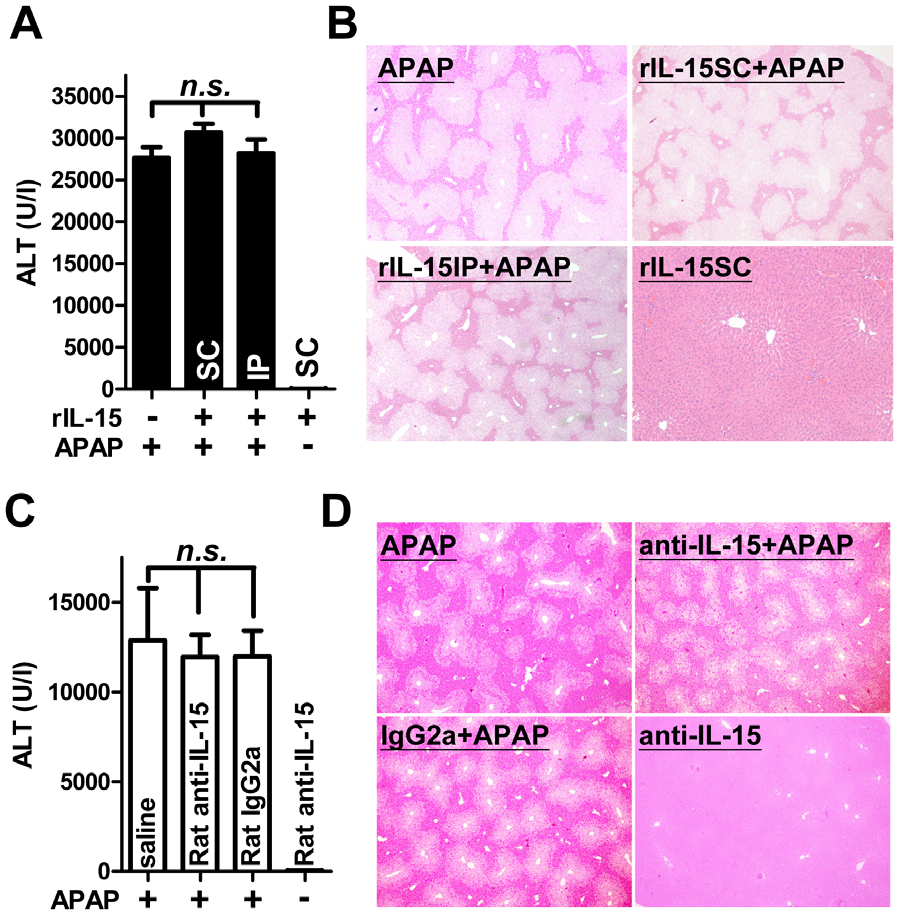
Effects of exogenous IL-15 or IL-15 neutralizing antibody administration on AILI in *Il15^−/−^* or WT mice. Before APAP injection, twenty micrograms of recombinant murine IL-15 were subcutaneously (SC) or intraperatoneally (IP) injected into *Il15^−/−^* mice. (A) Serum ALT levels and (B) representative liver histopathological changes at 8 hr after APAP injection (H&E staining; magnification ×4). One hundred and twenty micrograms of Rat anti-IL-15 neutralizing antibody or Rat IgG2a isotype control antibody were intraperitoneally injected into WT mice. (C) Serum ALT levels and (D) representative liver histopathological changes at 8 hr after APAP injection (H&E staining; magnification ×4). *n.s.*, not significant. Data represent means ± SEM from 5∼8 mice per group, and 3 mice in IL-15 SC and Rat anti-IL-15 neutralizing antibody only group.

The increased IL-15 levels in serum and KCs fraction during AILI in WT mice implicated its involvement in APAP hepatitis (Figure 2A,C). Previously, subcutaneous injection of recombinant IL-15 protected mice against concanavalin A-induced hepatitis [25]. Therefore, recombinant murine IL-15 was injected, subcutaneously or intraperitoneally, into *Il15^−/−^* mice to test the effect of the exogenous cytokine on APAP hepatotoxicity. Intriguingly, there was no significant difference among mice pretreated with recombinant IL-15, subcutaneously or intraperitoneally, or saline based on serum ALT level at 8 hr post-APAP (Figure 9A). In addition, hepatic histopathological examination showed unremarkable result among these groups (Figure 9B). Similarly, by effect assessments of serum ALT levels or hepatic histopathologies at 8 hr post-APAP (Figure 9C,D), pretreatment of WT mice with IL-15 neutralizing antibody did not change the APAP sensitivity. These results suggested that reconstitution or neutralization of IL-15 would not influence the susceptibility of mice to APAP, implicating a probable intracellular, but not extracellular, role of IL-15 within KC during AILI, or otherwise, a secondary phenomenon of elevated serum IL-15 in APAP hepatitis. In view of the up-regulated IL-15 expression in KC fraction of WT mice after APAP challenge (Figure 2C), we further examined the role of innate immunity in IL-15 protection against AILI.

### KCs but not neutrophils might play a major role in the heightened sensitivity to APAP in *Il15^−/−^* mice

**Figure 10:**
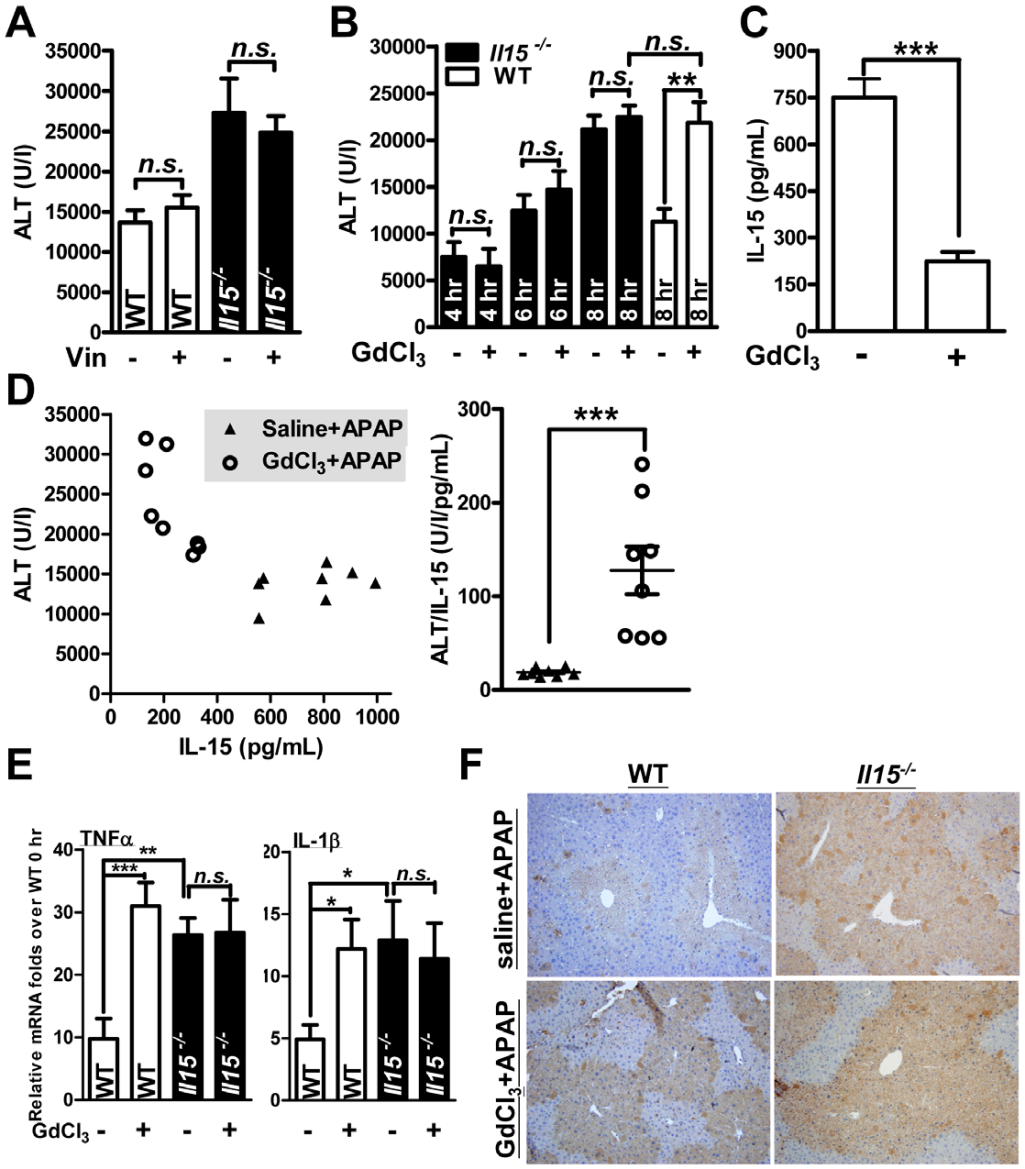
Effects of neutrophil and Kupffer cell elimination on AILI in mice. (A) 1.5 mg/kg vinblastine (Vin) was intravenously injected into mice 5 days before APAP challenge of mice. (B) Mice were intravenously injected with 30 mg/kg GdCl_3_ 36∼40 hr before APAP challenge. Serum ALT levels were analyzed at 4, 6 and 8 hr in *Il15^−/−^* mice, and 8 hr in WT mice after APAP injection. (C) The serum IL-15 levels, and (D) the dot plot of serum ALTs and relative IL-15 levels at 8 hr after APAP injection in WT mice with or without GdCl_3_ pretreatment. The ratio of ALT/IL-15 level was on the right panel. (E) Hepatic mRNA levels of TNFα and IL-1β and (F) hepatic nitrotyrosine formation (magnification ×20) at 8 hr post-APAP in WT and *Il15^−/−^* mice with GdCl_3_ pretreatment or not. *P<0.05; **P<0.01; ***P<0.001; *n.s.*, not significant. Data are mean ± SEM from 5∼8 mice per group.

Innate immune cells play an important role in APAP hepatitis [39]. To assay whether innate immune cells, such as neutrophils and KCs, are involved in exacerbated AILI of *Il15^−/−^* mice, we used vinblastine and GdCl_3_ for further experiments. Vinblastine, a mitosis inhibitor, was used to induce neutropenia in a mouse ischemia/reperfusion model [40]. Neutrophil depletion was confirmed by flow cytometry in mice (data not shown). Although increased neutrophil infiltration of liver was observed after APAP treatment in *Il15^−/−^* mice, pre-treatment of vinblastine could not alter the vulnerability of *Il15^−/−^* mice to AILI, based on the serum ALT levels at 8 hr post-APAP (Figure 10A). Similarly, vinblastine could not change the sensitivity to APAP hepatitis in WT mice (Figure 10A). The role of KCs in APAP hepatotoxicity still remained controversial [41], therefore we further evaluated the role of KCs in our model. A low dose of GdCl_3_ (7 mg/kg) was reported to inactivate KCs and modestly reduced APAP hepatitis [11], whereas the high dose of GdCl_3_ (30 mg/kg) has been reported to eliminate KCs in mice, thereby abrogating cadmium (Cd) induced hepatotoxicity [42]. Therefore, 30 mg/kg of GdCl_3_, a dose eliminating KCs by Indian ink uptake assay (data not shown) without liver damage, was used in our further studies. The KC-eliminated WT mice, by GdCl_3_, showed an increased susceptibility to APAP (Figure 10B,D), indicating a protective role of KCs in AILI and the accompanied inflammation (Figure 10E,F). This finding was compatible with previous protective role of KCs in AILI by Ju *et al.* in C57BL/6J WT mice [11]. Moreover, GdCl_3_ pre-treatment significantly reduced serum IL-15 elevation of WT mice during AILI (Figure 10C,D). Thus, KCs were probably the dominant IL-15 producer in APAP hepatitis. Since mice lack of KCs shared similar sensitivity to APAP hepatotoxicity with mice deficiency of IL-15 (Figure 10B), it implicated a regulatory correlation between IL-15 and KCs in AILI. Furthermore, elimination of KCs in *Il15^−/−^* mice did not affect the AILI severity (Figure 10B), hepatic TNFα and IL-1β (Figure 10E), as well as liver nitrotyrosine formation (Figure 10F), inferring a potential role of IL-15 in mediating KC effect on AILI. Further study is needed to clarify this assumption.

## Discussion

Use of genetically knockout mice is a novel strategy to elucidate the role of IL-15 in AILI. However, a recent report demonstrated the mispairing C57BL/6 controls of genetically engineered mice could lead to confounding results in AILI [43]. Moreover, this study showed the C57BL/6J substrains, which carried the nicotinamide nucleotide transhydrogenase (Nnt) mutation, were less susceptible to AILI than Nnt intact C57BL/6NJ (*Nnt^+/+^*) counterparts [43]. We also found that C57BL/6J but not C57BL/6NTac/*Il15^−/−^* mice carried Nnt mutation by DNA genotyping assay (Figure S4A). Therefore, C57BL/6NTac/*Il15^−/−^* mice were backcrossed to C57BL/6J substrains for 4 generations prior to further analysis of IL-15 effect on AILI. Similarly, we confirmed C57BL/6J (*Nnt^−/−^*) mice were less susceptible to APAP-hepatitis than C57BL/6NTac (*Nnt^+/+^*) counterparts, based on the 8-hr post-challenge serum ALT levels. Furthermore, C57BL/6NTac/*Il15^−/−^* or C57BL/6J/*Il15^−/−^* mice showed similarly higher susceptibility to APAP than their control counterparts (Figure S4B). Thus, *Nnt* or contamination of non-C57BL/6J substrain might not be involved in heightened APAP hepatotoxicity in *Il15^−/−^* mice of our study.

The efficacy of APAP metabolism (Figure 3) and the expressions of hepatic Nrf2-related and ROS detoxification genes (Figure 4) were similar in WT and *Il15^−/−^* mice. Therefore, the increased sensitivity of *Il15^−/−^* mice might not depend on liver parenchymal cells at the earlier stage of APAP toxicity. Indeed, after APAP challenge, we found productions of pro-inflammatory cytokines IL-1β, TNFα and IFNγ, as well as vascular adherence molecules and chemokines [6], [39], were greater in *Il15^−/−^* than WT mice (Figure 5), indicating their important roles in the increased APAP hepatotoxicity of *Il15^−/−^* mice.

Induction of iNOS, by pro-inflammatory cytokines such as IL-1β, IFNγ and TNFα in hepatocytes results in RNS formation [37], which further potentiates the APAP-induced hepatotoxicity [35]. It had been reported anti-oxidative proteins, Hsp70 and HO-1, protected mice against APAP hepatotoxicity [5], [36]. In a rat heat-shock model, Hoetzel *et al.* demonstrated that an NO donor could diminish HO-1 or Hsp70 induction by suppressing activator protein-1 (AP-1) DNA binding activity or modulating protein translation, respectively [44]. Our results in *Il15^−/−^* mice (Figure 6) suggested that excess hepatic iNOS and RNS production might potentiate APAP toxicity by suppressing HO-1 and Hsp70 induction (Figure 7,S3).

Through transcriptional arrest of hepatocytes, GalN with endotoxin or TNFα had been reported to induce acute apoptotic hepatitis in mice [45], whereas GalN pre-treatment markedly diminished the increased APAP hepatitis in *Il15^−/−^* mice versus a minor advantage in WT controls (Figure 8A–C). However, the apoptotic pathway was interrupted in our AILI model through mitochondria damage [46] and, moreover, GalN might block the secondary hepatic transcriptional activation from inflammatory mediators. In addition, the divergent responses between *Il15^−/−^* and WT mice to AILI might be partly attributed to the excess inflammation in the former. The detailed mechanism of GalN protection against APAP hepatitis needed further investigation. But, the protective effect of GalN was, at least, not through alteration of hepatic GSH contents in mice at early stage of AILI (Figure 8D).

Exogenous IL-15 administration into *Il15^−/−^* mice or IL-15 neutralizing antibody injection into WT counterparts could not alter the APAP hepatotoxicity (Figure 9), implicating the extracellular IL-15 was insufficient to affect the present fulminant AILI model. Moreover, increased serum IL-15 levels during AILI might be considered as the compensatory effect of hepatic inflammation and the activation of KCs. Although exogenous IL-15 did not affect APAP hepatitis, endogenous IL-15 seemed to play an interesting and pivotal role in AILI of our mice.

Prolonged innate inflammatory response induced by APAP-damaged hepatocyte determines the prognosis of liver injury [6]. APAP-induced inflammation was modulated by innate immune cells such as neutrophils [8], KCs [11] and DCs [9]. Neutrophil elimination did not protect against APAP hepatotoxicity in WT and *Il15^−/−^* mice (Figure 10A), which indicates a minor role of neutrophils in AILI [39]. Previously, KC produced hepato-protective factors (e.g., IL-10 and cyclooxygenase derived mediators) and depletion of KCs enhanced APAP hepatitis [11]. In ischemia-reperfusion (IR) liver injury, GdCl_3_ (30 mg/kg) protected mice via reduction of TNFα and IFNγ levels in liver effluents of balb/c mice [47], indicating the inflammatory contribution of KCs. However, Jaeschke *et. al.* [39] suggested the protective role of KCs, but not inflammatory effecter, during AILI, and hepatic inflammation might be enhanced by other NPCs, such as endothelial cells [19], [39]. During AILI, cyclooxygenase products from KCs not only suppressed inflammation but also up-regulated HSPs [39], whereas enhanced hepatic inflammation was found in APAP-injected *Il15^−/−^* mice, with decreased HSP induction (Figure 5, 7, S3). Additionally, elimination of KCs by GdCl_3_ did not alter the severity of AILI in *Il15^−/−^* mice, but increased the APAP sensitivity of WT mice, leading to a similar susceptibility in both groups of mice (Figure 10B). Moreover, pretreatment of WT mice with GdCl_3_ diminished serum IL-15 elevation during AILI (Figure 10C) and showed a negative correlation between serum IL-15 level and liver damage extent (Figure 10D). Furthermore, with GdCl_3_ pretreatment, hepatic TNFα, IL-1β and nitrotyrosine levels were up-regulated in WT mice, indicating the inflammation prevention by KCs in our model (Figure 10E,F). However, depletion of KCs did not affect the enhanced hepatic inflammation in *Il15^−/−^* mice after APAP challenge (Figure 10E,F). These findings suggested the probable upstream and downstream relationship of KCs and IL-15 in the protection against AILI. IL-15 had been found to determine the activation level of macrophages in mouse rheumatoid arthritis model [48]. In addition, IL-15, at extremely low concentration, selectively suppresses pro- (e.g., TNFα, IL-6 and IL-1) but not anti-inflammatory (e.g., IL-10) cytokine production in LPS-activated macrophage [24]. Thus, IL-15 might potentially influence KC function and thereby modulate the inflammatory response during AILI.

There were two isoforms of IL-15, short and long signal peptide isoforms, existing in human and murine cells [49], [50], [51], [52]. After translation, the short (intracellular) IL-15 isoform was not secreted but rather distributed within the cytoplasm and the nucleus [52]. Although the biological function of intracellular IL-15 had not been fully elucidated in KCs, it could function as a histone deacetylase inhibitor regulating the expression of IL-12 receptor β1 in macrophage [53]. Moreover, intracellular IL-15 could indirectly regulate mMCP-2 induction by altering the expressions of transcription factors C/EBPβ and YY1 in mouse mast cells [54]. Therefore, based on previous reports and our results, IL-15 might function uniquely within KCs in AILI.

In previous studies, IL-15 was found to enhance severity of endotoxin shock liver injury [27], colitis [55] and virus infection [56]. In contrast, IL-15 protected against nephrotoxic serum nephritis [57] and experimental autoimmune encephalomyelitis [58]. Increased serum IL-15 level was also noted in patients with liver transplantation [59] or hepatitis C virus infection [60]. Here, we demonstrated an enhanced APAP susceptibility in *Il15^−/−^* mice, which might result from an overactive inflammatory response. In view of these results, further studies might be justified to elucidate the biological function of intracellular IL-15 within KCs during AILI.

## Supporting Information

Figure S1 **The induction of pro-inflammatory cytokines in serums and livers after APAP challenge in mice.** (A) The serum levels of IL-1β, TNFα and IFNγ and (B) hepatic protein levels of IL-1β and IFNγ at 8 hr after treatment with APAP. *P<0.05; **P<0.01; ***P<0.001. Data are mean ± SEM from 6∼8 mice per group.
(TIF)

Figure S2 **The number of infiltrated hepatic neutrophils is greater in** ***Il15^−/−^*** **mice after APAP injection.** Hepatic non-parenchymal cells were isolated from APAP-injected WT and *Il15^−/−^* mice. Cells were stained for CD11b and F4/80 cell surface markers. CD11b+ cells were gated to demonstrate in dot plots. (A) The relative percentages of neutrophils and KCs in CD11b+ cells at 0 and 8 hr after APAP injection, dot plots and numbers are the representative data. (B) Data are from 3∼5 mice at 0 and 8 hr, respectively, after APAP challenge. *P<0.05. Data are mean ± SEM from 3∼5 mice per group.
(TIF)

Figure S3 **The level of hepatic Hsp70 is lower in** ***Il15^−/−^*** **mice after APAP challenge.** The Hsp70/HSC70 levels were evaluated by quantitative PCR and western blot analysis. mRNA levels of (A) Hsp70 and (B) HSC70, and (C) protein levels and quantification data of Hsp70/HSC70 at 8 hr post-APAP injection in mice. #*P*<0.05, compared with WT at 0 hr; *P<0.05; **P<0.01. Data are mean ± SEM from 5∼8 mice per group.
(TIF)

Figure S4 **Effect of Nnt mutation on APAP-induced hepatitis in WT or** ***Il15^−/−^*** **mice.** (A) Nnt genotyping results of WT and *Il15^−/−^* mice. DNA samples from mice were evaluated with *Nnt^+/+^* (wt) and *Nnt^−/−^* (mut) genetic markers by PCR. (B) Serum levels of ALT at 8 hr after APAP treatment in WT and *Il15^−/−^* mice. *P<0.05; **P<0.01; **P<0.001. Data are mean ± SEM from 5∼8 mice per group.
(TIF)

Methods S1 **Determination of NPC sub-population with flow cytometry, and** ***Nnt*** **genotyping of the mice.**
(DOC)

Table S1 **Oligonucleotide Sequences Used for Real-time PCR or genotyping.**
(DOC)
