# Supplementary figures and images for: Deficiency of Interleukin-15 Enhances Susceptibility to Acetaminophen-Induced Liver Injury in Mice
Source: PLoS One. 2012 Sep 18;7(9):e44880. doi: 10.1371/journal.pone.0044880 (PMC3445599; doi:10.1371/journal.pone.0044880)

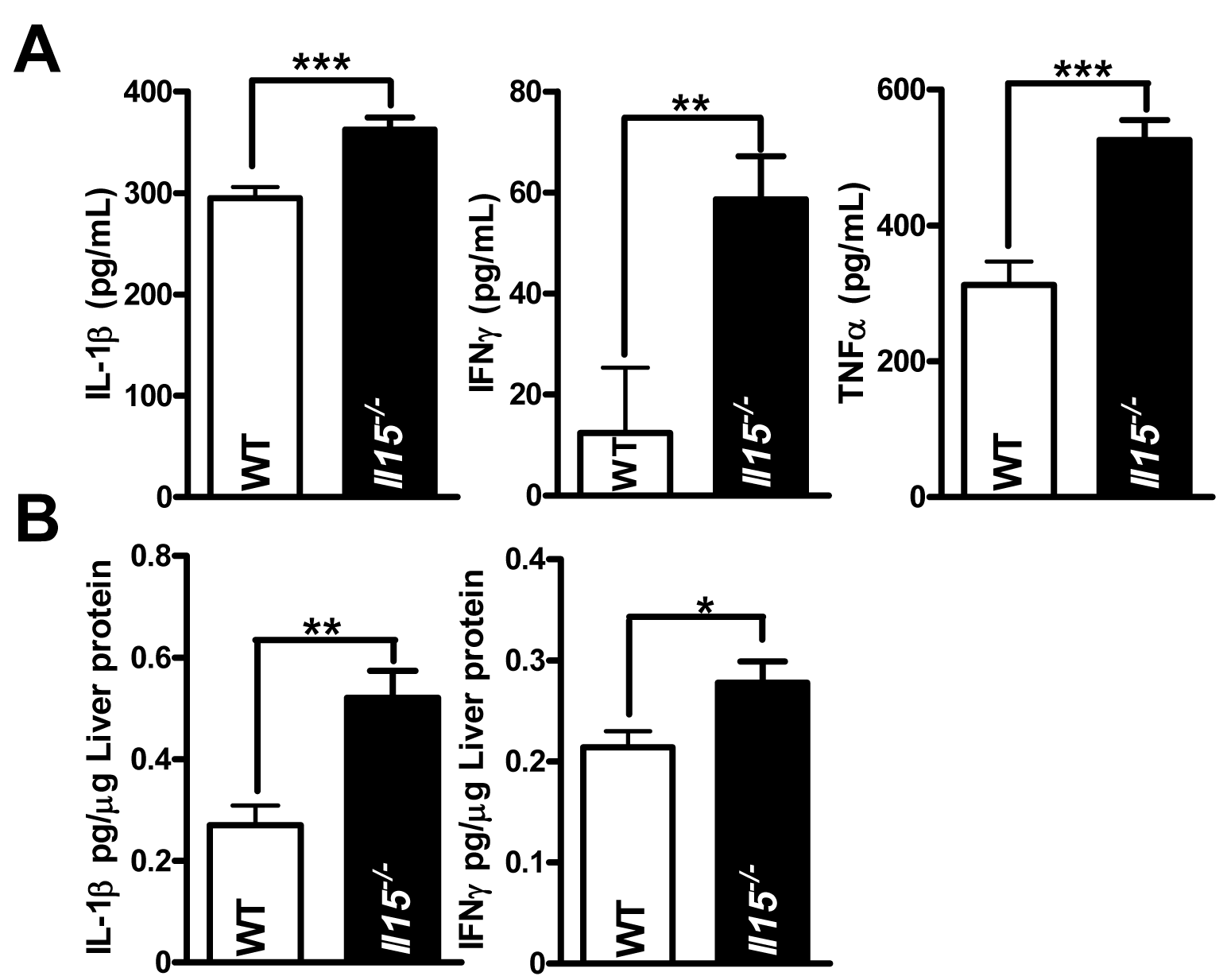

Supplement: Figure S1 — The induction of pro-inflammatory cytokines in serums and livers after APAP challenge in mice. (A) The serum levels of IL-1β, TNFα and IFNγ and (B) hepatic protein levels of IL-1β and IFNγ at 8 hr after treatment with APAP. *P<0.05; **P<0.01; ***P<0.001. Data are mean ± SEM from 6∼8 mice per group. (TIF) [file pone.0044880.s001.tif]

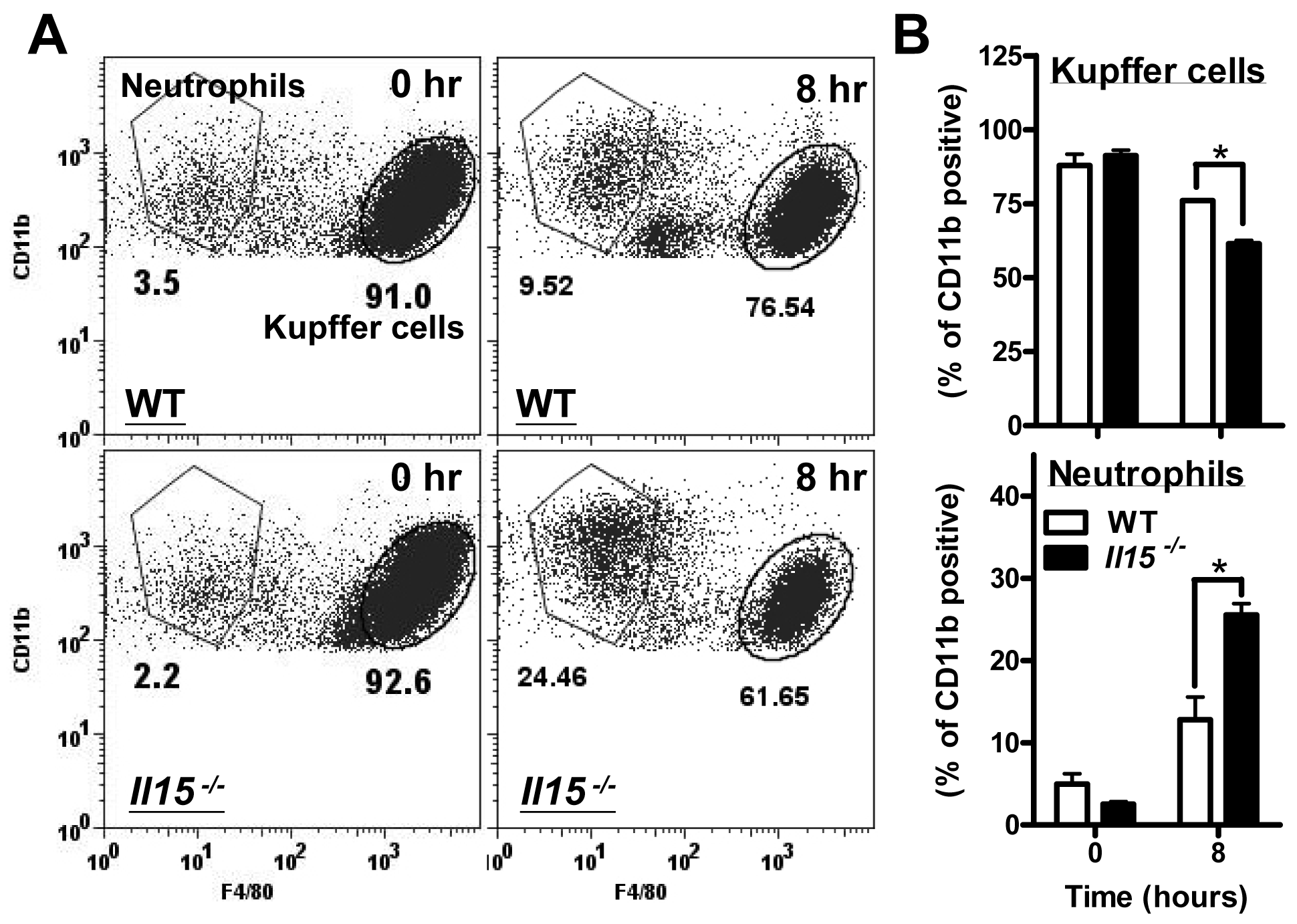

Supplement: Figure S2 — The number of infiltrated hepatic neutrophils is greater in Il15−/− mice after APAP injection. Hepatic non-parenchymal cells were isolated from APAP-injected WT and Il15−/− mice. Cells were stained for CD11b and F4/80 cell surface markers. CD11b+ cells were gated to demonstrate in dot plots. (A) The relative percentages of neutrophils and KCs in CD11b+ cells at 0 and 8 hr after APAP injection, dot plots and numbers are the representative data. (B) Data are from 3∼5 mice at 0 and 8 hr, respectively, after APAP challenge. *P<0.05. Data are mean ± SEM from 3∼5 mice per group. (TIF) [file pone.0044880.s002.tif]

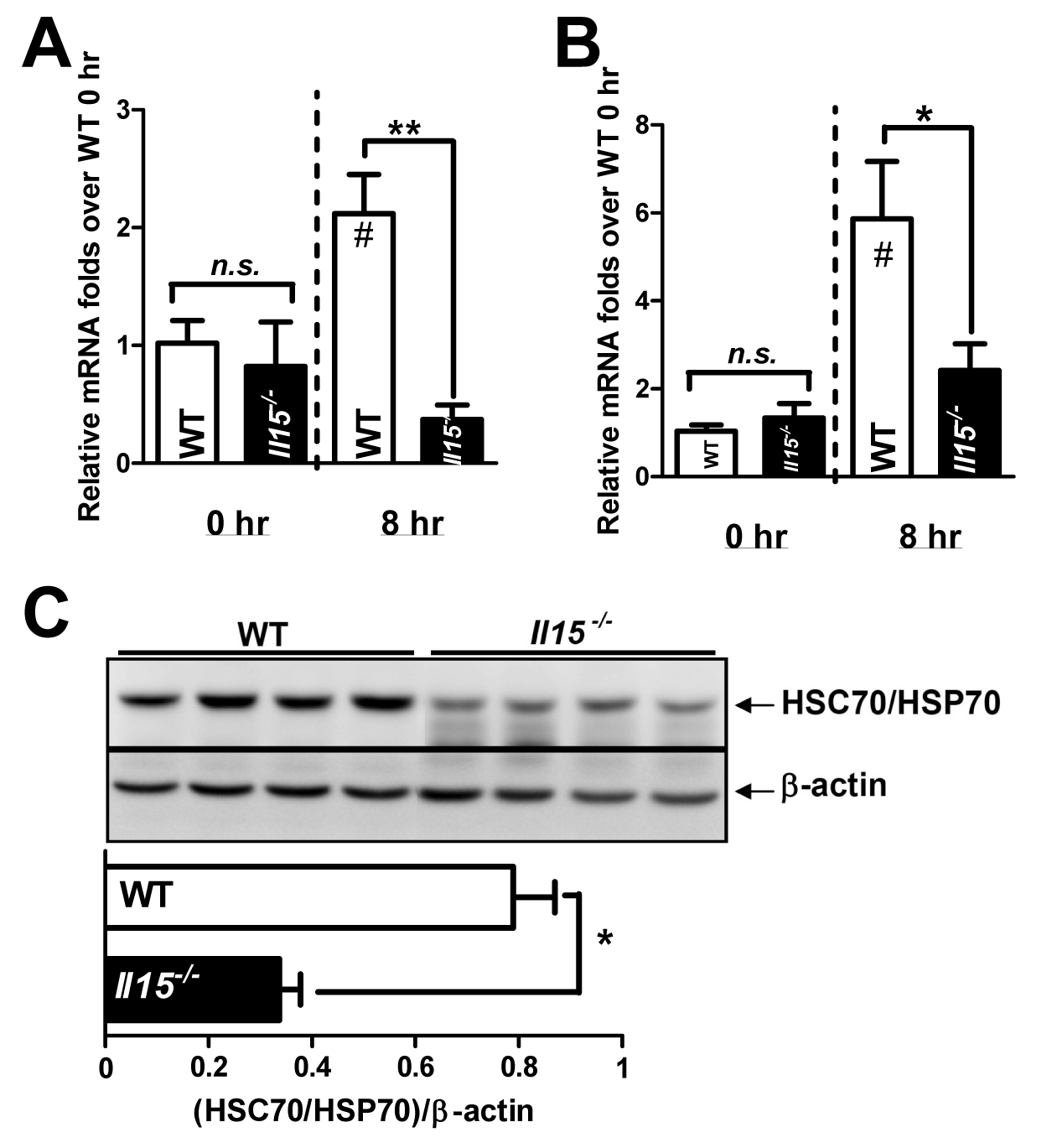

Supplement: Figure S3 — The level of hepatic Hsp70 is lower in Il15−/− mice after APAP challenge. The Hsp70/HSC70 levels were evaluated by quantitative PCR and western blot analysis. mRNA levels of (A) Hsp70 and (B) HSC70, and (C) protein levels and quantification data of Hsp70/HSC70 at 8 hr post-APAP injection in mice. #P<0.05, compared with WT at 0 hr; *P<0.05; **P<0.01. Data are mean ± SEM from 5∼8 mice per group. (TIF) [file pone.0044880.s003.tif]

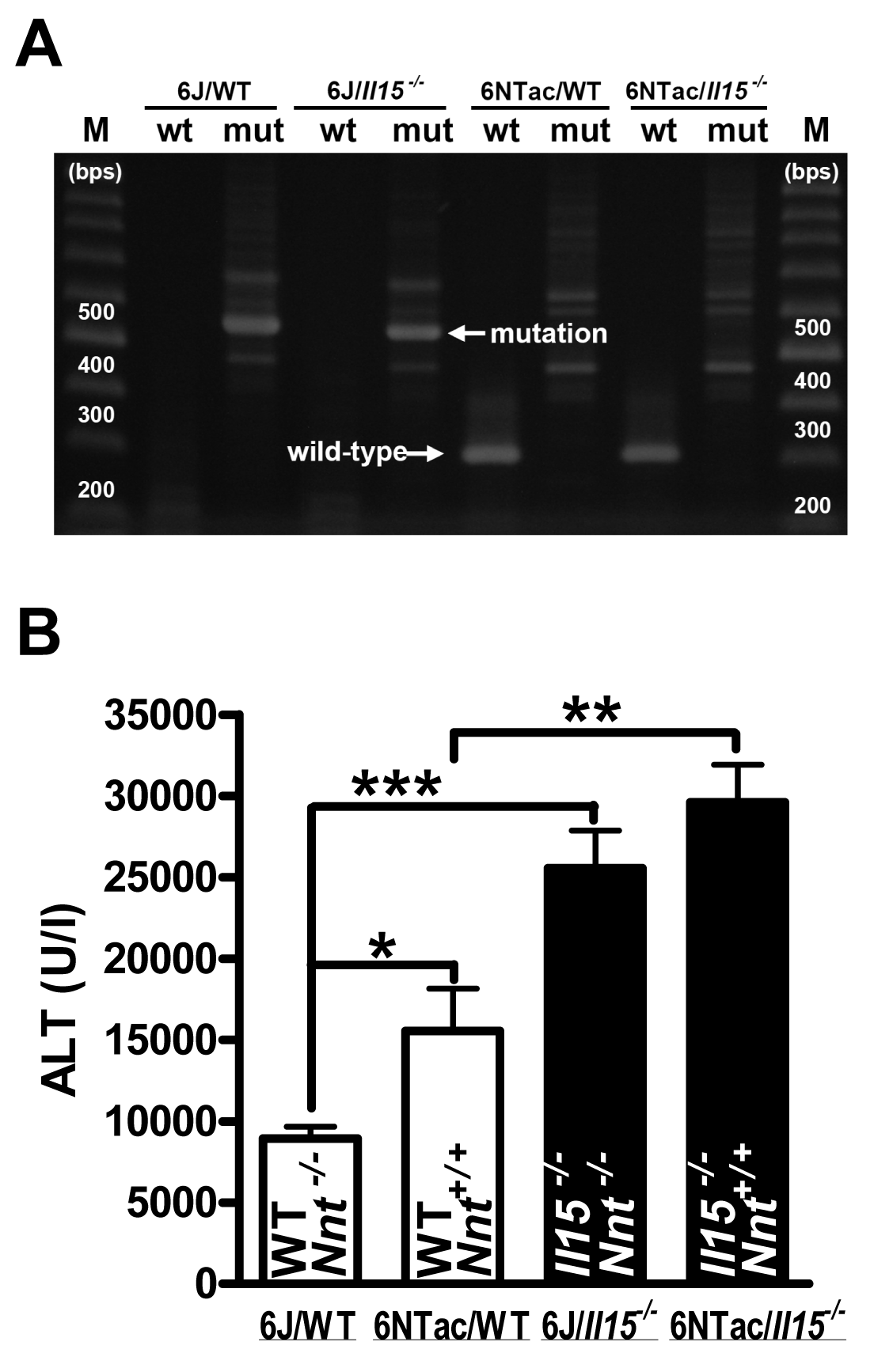

Supplement: Figure S4 — Effect of Nnt mutation on APAP-induced hepatitis in WT or Il15−/− mice. (A) Nnt genotyping results of WT and Il15−/− mice. DNA samples from mice were evaluated with Nnt+/+ (wt) and Nnt−/− (mut) genetic markers by PCR. (B) Serum levels of ALT at 8 hr after APAP treatment in WT and Il15−/− mice. *P<0.05; **P<0.01; **P<0.001. Data are mean ± SEM from 5∼8 mice per group. (TIF) [file pone.0044880.s004.tif]
