# Supplementary material for: Deficiency of Interleukin-15 Enhances Susceptibility to Acetaminophen-Induced Liver Injury in Mice
Source: PLoS One. 2012 Sep 18;7(9):e44880. doi: 10.1371/journal.pone.0044880 (PMC3445599; doi:10.1371/journal.pone.0044880)
Supplement: Methods S1 — Determination of NPC sub-population with flow cytometry, and Nnt genotyping of the mice. (DOC) [file pone.0044880.s005.doc]

**Supplementary Methods:**

***Determination of NPC sub-population with flow cytometry.*** NPCs were characterized as previously described [1] with minor modification to analyze the composition of the hepatic-infiltrated mononuclear cells. The anti-mouse CD16/32 antibody BD Biosciences (San Jose, CA, USA) was used to block non-specific binding in isolated NPCs, which were then stained with antibodies to detect the population of neutrophils and KCs in livers: fluorescein isothiocyanate (FITC)-labeled anti-CD45, FITC-labeled anti-Gr1, phycoerythrin-lebeled anti-CD11b and allophycocyanin (APC)-labeled anti-F4/80 (eBiscience). Flow cytometry involved use of a FACS Calibur cytometer (Becton Dickinson Immunocytometry Systems, San Jose, CA, USA).

***Nicotinamide nucleotide transhydrogenase (Nnt) genotyping***. Genomic DNA was purified from mouse tail and Nnt genotype was characterized by use of PCR. Two sets of primers have been previously published [2] and listed in Supporting Information Table S1.

**References**

1. Holt MP, Cheng L, Ju C (2008) Identification and characterization of infiltrating macrophages in acetaminophen-induced liver injury. J Leukoc Biol 84: 1410-1421.

2. Huang TT, Naeemuddin M, Elchuri S, Yamaguchi M, Kozy HM, et al. (2006) Genetic modifiers of the phenotype of mice deficient in mitochondrial superoxide dismutase. Hum Mol Genet 15: 1187-1194.
