# Supplementary material for: Deficiency of Interleukin-15 Enhances Susceptibility to Acetaminophen-Induced Liver Injury in Mice
Source: PLoS One. 2012 Sep 18;7(9):e44880. doi: 10.1371/journal.pone.0044880 (PMC3445599; doi:10.1371/journal.pone.0044880)
Supplement: Table S1 — Oligonucleotide Sequences Used for Real-time PCR or genotyping. (DOC) [file pone.0044880.s006.doc]

Table S1. Oligonucleotide Sequences Used for Real-time PCR or genotyping

| Gene | Sequence (5’ – 3’) | mRNA accession ID  PCR product size |
| --- | --- | --- |
| GAPDH | Forward : accacagtccatgccatcac  Reverse : gggatgatgttctgggcagc | NM_008084.2  98 bps |
| CYP2E1 | Forward : gtctcctcatagagatggagaagg  Reverse : gtcggccaaagtcacagaa | NM_021282.2  83 bps |
| CYP1A2 | Forward: cctggactgactcccacaac  Reverse: cgccatctgtaccactgaag | NM_009993.3  63 bps |
| GCLC | Forward: gatgatagaacacgggaggaga  Reverse: tgatcctaaagcgattgttcttc | NM_010295.1  61 bps |
| GstPi | Forward: tgtcaccctcatctacaccaac  Reverse: gacagcagggtctcaaaagg | NM_013541.1  93 bps |
| NQO-1 | Forward: agcgttcggtattacgatcc  Reverse: agtacaatcagggctcttctcg | NM_008706.5  68 bps |
| MRP-2 | Forward : caaatccaattctctacctatgcac  Reverse : gcctgcagtgttggatca | NM_013806.2  60 bps |
| MRP-3 | Forward: gctgagggtggggataatct  Reverse: ggctcgggctaggcatac | NM_029600.3  60 bps |
| SOD-1 | Forward: caggacctcattttaatcctcac  Reverse: tgcccaggtctccaacat | NM_011434.1  78 bps |
| SOD-2 | Forward: gacccattgcaaggaacaa  Reverse: gtagtaagcgtgctcccacac | NM_013671.3  69 bps |
| catalase | Forward: ccttcaagttggttaatgcaga  Reverse: caagtttttgatgccctggt | NM_009804.2  80 bps |
| IL-1β | Forward: tgagcaccttcttttccttca  Reverse: gcagctgtctaatgggaacg | NM_008361.3  105 bps |
| IL-6 | Forward: gaggataccactcccaacagacc  Reverse: aagtgcatcatcgttgttcataca | NM_031168.1  141 bps |
| IL-15 | Forward: gaggaatacatccatctcgtgc  Reverse: cctacactgacacagcccaaaa | NM_008357.1  102 bps |
| TNFα | Forward: tcttctcattcctgcttgtgg  Reverse: ggtctgggccatagaactga | NM_013693.2  128 bps |
| VCAM-1 | Forward: tggtgaaatggaatctgaacc  Reverse: cccagatggtggtttcctt | NM_011693.3  86 bps |
| ICAM-1 | Forward: cccacgctacctctgctc  Reverse: gatggatacctgagcatcacc | NM_010493.2  72 bps |
| MIP-1α | Forward: tgcccttgctgttcttctct  Reverse: gtggaatcttccggctgtag | NM_011337.2  113 bps |
| MIP-2α | Forward: aaaatcatccaaaagatactgaacaa  Reverse: ctttggttcttccgttgagg | NM_009140.2  91 bps |
| KC/GRO | Forward: tcgccaatgagctgcgctgtc  Reverse: gcttcagggtcaaggcaagcc | NM_008176.3  160 bps |
| NO-1 | Forward: aggctaagaccgccttcct  Reverse: tgtgttcctctgtcagcatca | NM_010442.2  72 bps |
| Hsp70 | Forward: ggccagggctggattact  Reverse: gcaaccaccatgcaagatta | NM_010479.2  73 bps |
| Hsc70 | Forward: ggaatacaaaggggagacaaaa  Reverse: acagcgttggtaacggtctt | NM_031165.4  113 bps |
| Nnt^+/+^ | Forward: caattctgccaacaactgga  Reverse: ggtcactctgggcactgttt | 312 bps |
| Nnt^-/-^ | Forward: gtagggccaactgtttctgc  Reverse: tcccctcccttccatttagt | 547 bps |

GAPDH, glyceraldehyde 3-phosphate dehydrogenase; CYP2E1, cytochrome P450 2E1; CYP1A2, cytochrome P450 1A2; GCLC, glutamate-cysteine ligase, catalytic subunit; GstPi-1, glutathione S-transferase pi 1; NQO-1, NAD(P)H:quinone oxidoreductase 1; MRP-2, multidrug resistance protein 2; MRP-3, multidrug resistance protein 3; SOD-1, superoxide dismutase-1; SOD-2, superoxide dismutase-2; IL-1β, interleukin-1beta; IL-6, interleukin-6; IL-15, interleukin-15; TNFα, tumor necrosis factor alpha; ICAM-1, intercellular adhesion molecule-1; VCAM-1, vascular cell adhesion protein-1; MIP-1α, macrophage inflammatory protein-1 alpha; MIP-2α, macrophage inflammatory protein-2 alpha; KC/GRO, KC/growth-regulated alpha protein; HO-1, hemeoxygenase 1, Hsp70, heat shock protein 70, HSC70, heat shock protein cognate 70. Nnt, nicotinamide nucleotide transhydrogenase.
